# Analysis of Salivary Cytokines in Retinopathy of Prematurity

**DOI:** 10.3390/children12010080

**Published:** 2025-01-10

**Authors:** Hwa-Shiu Wu, Hsin-Chun Huang, I-Lun Chen

**Affiliations:** 1 Department of Pediatrics, Kaohsiung Chang Gung Memorial Hospital, College of Medicine, Chang Gung University, Kaohsiung 83301, Taiwan; s10101010@cgmh.org.tw (H.-S.W.); hhuang@cgmh.org.tw (H.-C.H.); 2 School of Traditional Chinese Medicine, College of Medicine, Chang Gung University, Linkou 33302, Taiwan

**Keywords:** retinopathy of prematurity, saliva, cytokines

## Abstract

Background/Objectives: This cohort study aimed to establish a correlation between salivary cytokines and retinopathy of prematurity (ROP) in premature neonates. Additionally, we sought to identify a minimally invasive method for cytokine detection in this population. Methods: We recruited premature neonates born at less than 34 weeks gestational age (GA), with no history of maternal or neonatal infections. Salivary samples were collected on their first (D1) and seventh (D7) days of life, and cytokine levels were measured using the MILLPLEXMAP Human multiplex assay. Results: A total of 125 neonates were included in the study, categorized into two groups based on the severity of ROP: None to Mild and Moderate to Severe. The salivary levels of interleukin (IL)-6, IL-8, vascular endothelial growth factor (VEGF), and tumor necrosis factor (TNF)-α on D1 and D7 were significantly higher in the Moderate to Severe ROP group compared to the None to Mild ROP group (*p* = 0.005, 0.004, 0.026, 0.018, 0.001, 0.007, 0.025, and 0.002, respectively). After adjusting for GA, the levels of IL-6 and VEGF on D7 were significantly elevated in the Moderate to Severe ROP group compared to the None to Mild ROP group (*p* = 0.024 and 0.016, respectively). Conclusions: This study establishes a novel, non-invasive method for the early prediction of ROP in premature neonates by correlating salivary cytokine levels in early life with the subsequent development of ROP.

## 1. Introduction

Retinopathy of prematurity (ROP) is a leading cause of blindness in children born prematurely worldwide [1,2]. Approximately 10% of premature infants are affected by ROP, with severity increasing in relation to decreasing gestational age (GA) and birth weight [3]. The development of ROP is influenced by multiple factors, primarily related to the stress of perinatal oxygen metabolism. Additional risk factors, such as sepsis, nutritional deficiencies, and necrotizing enterocolitis, can also further increase the risk of ROP. These conditions contribute to oxidative stress and inflammation, which impair the normal formation and maturation of retinal vasculature [4]. Angiogenic factors, such as Vascular Endothelial Growth Factor (VEGF), and inflammatory response-related cytokines, including interleukin-6 (IL-6), interleukin-8 (IL-8), and tumor necrosis factor-alpha (TNF-α), have been reported to play significant roles in this process [4,5,6]. Clinically, ROP is asymptomatic in its early stages, and diagnosis relies primarily on retinal examinations conducted by experienced ophthalmologists when the infant is between 4 and 9 weeks old. The early detection and management of ROP are critical for reducing the risk of blindness [7].

Recent studies have focused on understanding how VEGF contributes to the development of ROP, revealing that vitreous VEGF levels increase as ROP progresses [8,9,10]. Additionally, several biomarkers, such as metabolites, inflammatory cytokines, growth factors, gut microbiota, and noncoding RNAs, have been linked to ROP, with higher serum levels of inflammatory cytokines observed in ROP patients compared to those without ROP [11,12,13]. While some research has shifted toward analyzing cytokines in the aqueous humor of premature infants, providing valuable insights, the invasive nature of such procedures can cause significant stress to the infants [14].

Given that ROP predominantly affects preterm infants, the invasive collection of blood or aqueous fluid can be detrimental and may have long-lasting effects. Consequently, recent studies have explored non-invasive sample collection methods, such as using amniotic fluid or umbilical cord blood, to predict ROP [15,16,17]. However, these approaches often fail to allow for the continuous monitoring of cytokine levels after birth, limiting the ability to observe changes associated with ROP development.

Tear fluid cytokines have been investigated for their correlation with ROP occurrence, although tears are less convenient to collect than saliva [18]. Saliva can be obtained more easily and quantified more effectively. Our previous studies, along with others, have demonstrated a correlation between cytokine levels in saliva and blood [19,20,21], and we have also found that salivary cytokines in preterm infants can predict bronchopulmonary dysplasia (BPD) [22]. Therefore, there is a critical need for a more accessible predictive model that leverages these biomarkers to facilitate the early detection and prediction of ROP. This study was conducted to explore the correlation between changes in salivary cytokine concentrations of preterm infants and the development of ROP.

## 2. Materials and Methods

### 2.1. Study Design and Patient Population

This prospective cohort study was conducted with approval from the Institutional Review Board (IRB) of Chang Gung Memorial Hospital (IRB numbers: 202401181B0). Patients born between August 2012 and May 2017 who met the inclusion criteria were enrolled. Eligible premature infants had a birth weight of 2000 g or less, a GA of 34 weeks or less, and required retinal examination for ROP. Exclusion criteria included infants born to mothers with bacteremia or clinical chorioamnionitis (indicated by intrapartum fever, uterine tenderness, or malodorous amniotic fluid, and confirmed by bacterial growth in amniotic fluid or placenta cultures), critical congenital heart malformations, lung anomalies, congenital sepsis or pneumonia (identified by positive blood, sputum, or urine cultures within the first week of life), and congenital Group B Streptococcus (GBS) infection (confirmed by a positive urine GBS antigen or culture). Infants with other prevalent congenital viral infections (determined by positive neonatal or maternal serum IgM for pathogens such as toxoplasma, rubella, cytomegalovirus (CMV), herpes simplex virus, etc.) were also excluded. Additionally, infants who died before their first discharge were omitted from the study.

The feeding strategies of these included babies were based on the recommendations of the Nutrition Care of Taiwan Preterm Infants. Parenteral nutrition with 2–3 g/kg amino acid was started on the first day of life. Glucose infusion rate 4–8 mg/kg/min was administered according to the blood sugar level. Enteral feeding began within 24–48 h of life unless the vital signs were unstable.

### 2.2. Salivary Collection

Salivary specimens were collected from all participants within 24 h after birth (D1) and on their seventh day of life (D7). On the first day of life, these preterm infants were in a “nothing by mouth” (NPO) status, receiving continuous intravenous fluid with 10% glucose water or parenteral nutrition. By the seventh day, some neonates had commenced minimal feeding via a nasogastric tube. Total parenteral nutrition and lipid emulsions were administered in accordance with NICU protocols. To minimize the potential impact of feeding and diurnal rhythms on this study, saliva samples were consistently collected before feeding around 7:00 AM on the seventh day of life [23].

The neonates were positioned supine in the incubator or warmer, with an 8 Fr suction catheter placed in the mouth. A negative pressure machine was activated to collect a minimum of 0.5 mL of saliva in a sputum collection box. Samples were immediately placed in an icebox and transported to the laboratory, where they were pipetted into 1.5 mL sterile Eppendorf tubes and stored at −80 °C until batch analysis [21]. The procedure would be immediately discontinued if any abnormal vital signs were detected, such as a heart rate below 100 bpm, a mean blood pressure lower than the corrected gestational age, oxygen saturation below 80%, or a respiratory rate exceeding 80 breaths per minute, regardless of the duration of the abnormality. All these parameters were continuously displayed on the monitor.

### 2.3. Patient Grouping and Data Collection

Diagnosis and classification of ROP were based on the International Classification of Retinopathy of Prematurity (ICROP) [24]. An experienced ophthalmologist performed diagnostic examinations using an indirect ophthalmoscope (Vantage Plus, Keeler, Windsor, UK). The initial ROP examination was performed 4 to 9 weeks after birth or between the 31st and the 33rd weeks of gestation. Follow-up examinations were conducted at 2- to 3-week intervals based on initial retinal findings. The classification of ROP adhered to guidelines provided by the American Academy of Pediatrics and the American Association for Pediatric Ophthalmology [7]. Mydriasis was induced using either tropicamide 0.5% or phenylephrine 2.5% before ophthalmoscopic examinations. ROP severity was classified into five stages, from mild (stage 1) to severe (stage 5). The patients were grouped based on the most severe stage of ROP identified during initial and subsequent examinations. Infants with no ROP (stage 0) and mild ROP (stage 1) were placed in the None to Mild ROP group, while those with stage 2 or above ROP were assigned to the Moderate to Severe ROP group and were designated for close follow-up or further treatment. Clinical characteristics, including GA, birth weight, sex, mode of delivery, Apgar scores, vital signs, and the duration of intubation during hospitalization, were collected from electronic medical records. Comorbidities were also documented, including intraventricular hemorrhage (IVH), periventricular leukomalacia (PVL), BPD (based on guidelines of the National Institutes of Child Health and Human Development (NICHD)) [25], and necrotizing enterocolitis (NEC), as outlined in our previous study [22]. Maternal characteristics such as gravida/para status, the duration of prolonged rupture of membranes, preeclampsia, gestational diabetes mellitus (GDM), and antenatal fever and medication were also included.

### 2.4. Cytokine Analysis

Salivary cytokines, including IL-6, IL-8, TNF-α, and VEGF were measured using the MILLIPLEX^®^ MAP Human multiplex assay according to the manufacturer’s instructions. The assay involved mixing 25 μL of salivary samples with antibody-conjugated beads, which were then analyzed using a multi-channel system, as detailed in our previous study [22]. A Luminex instrument (Austin, TX, USA) was used to run the plates and generate quantitative data. Fluorescence data were captured and analyzed using MasterPlex^TM^ QT software (ver. 1.2; MiraiBio, Inc., South San Francisco, CA, USA). Cytokine concentrations were calibrated by interpolating a series of standard samples as recommended by the manufacturer.

### 2.5. Statistics

Continuous data were analyzed using the Mann–Whitney *U* test, while categorical data were assessed using chi-squared or Fisher’s exact test. Linear regression was employed to estimate the correlation between GA and cytokines levels. Additional analyses were conducted using a multivariable logistic regression model. All statistical analyses were performed using SPSS Statistics software (version 25.0, IBM Corp., Armonk, NY, USA), considering a *p*-value of <0.05 as statistically significant. Cases with missing data were excluded pairwise during statistical analysis.

## 3. Results

### 3.1. Patient Enrollment

Patient enrollment followed the protocol established in our previously published study [22]. Initially, 186 patients were recruited; however, 43 were subsequently excluded due to maternal or neonatal infections. The exclusions included 2 cases of maternal sepsis, 33 cases of maternal chorioamnionitis, 1 case of CMV infection, 2 cases of neonatal sepsis, 2 cases of congenital pneumonia, and 3 cases of neonatal GBS infection. Of the remaining patients, 18 neonates passed away before their first discharge, resulting in their exclusion from the study.

**Figure 1:**
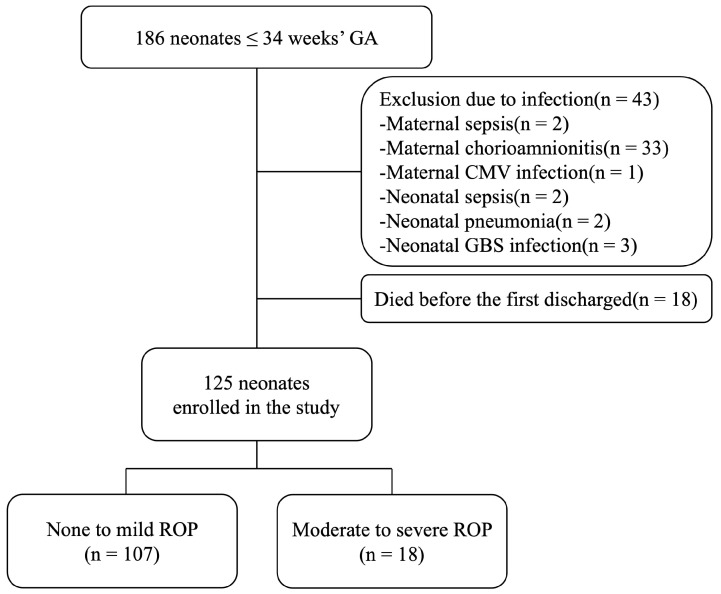
Patient enrollment. A total of 186 neonates born at or less than 34 weeks’ GA were recruited. Of these, 43 were excluded due to infections, and 18 neonates died before discharge. Ultimately, 125 surviving neonates were enrolled and categorized into two groups based on the severity of their ROP.

Ultimately, 125 premature infants who survived and met the inclusion criteria were enrolled. These infants were categorized into two groups: 108 in the None to Mild ROP group and 17 in the Moderate to Severe ROP group (Figure 1). All 125 premature infants were cared for in our NICU or sick baby unit until they reached at least 36 weeks of postmenstrual age (PMA) or were deemed ready for discharge by a neonatologist. All patients underwent at least one ROP screening before discharge and continued follow-up at our outpatient ophthalmology department, with follow-up intervals determined by the ophthalmologists.

### 3.2. Patient Demographics

The demographic data of the patients are presented in Table 1. Significant inverse correlations were observed between the severity of ROP and both neonatal GA and birth weight (both *p* < 0.001). Additionally, the number of days of intubation and the presence of BPD were positively associated with the severity of ROP (both *p* < 0.001). No significant differences were found between the two groups in terms of sex, mode of delivery, initial vital signs, IVH, or PVL. Furthermore, there were no statistically significant differences in maternal conditions, including preeclampsia, GDM, antenatal fever, or medication use.

### 3.3. Salivary Cytokines

**Figure 2:**
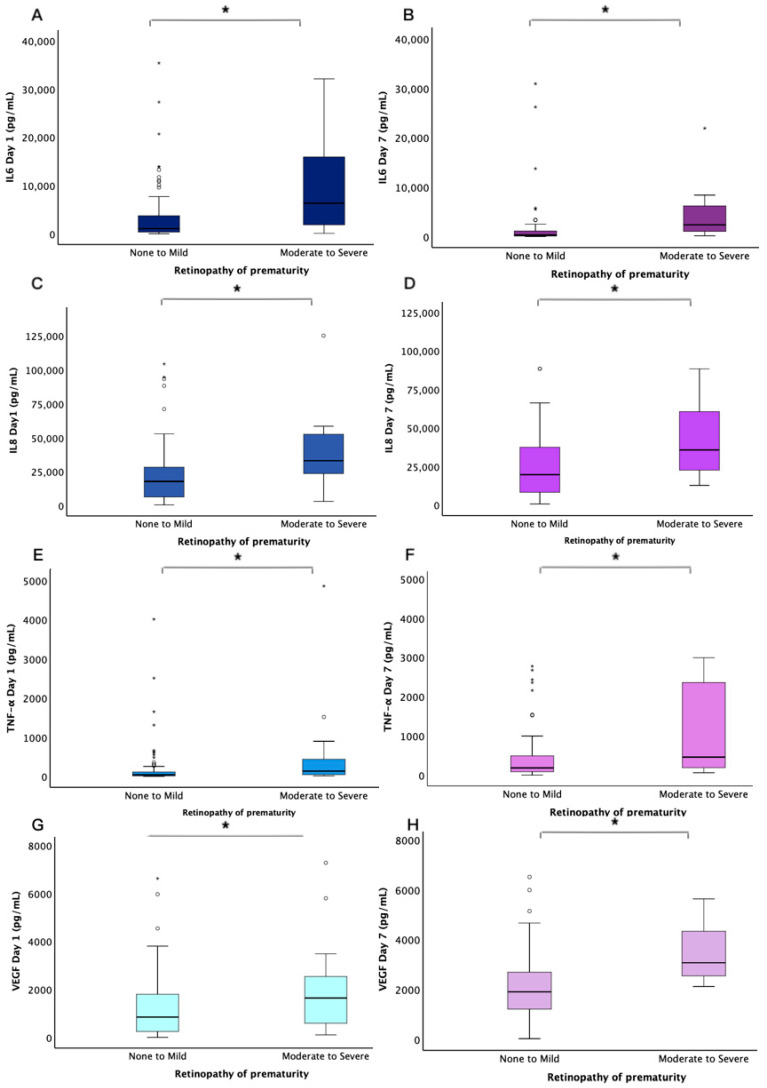
Salivary cytokine levels in neonates with varying ROP severity. The blue boxplots represent cytokine concentrations in the two groups on D1 (**A**,**C**,**E**,**G**), while the purple boxplots represent concentrations on D7 (**B**,**D**,**F**,**H**). The horizontal black lines within the boxes indicate the median cytokine levels. Asterisks denote significant differences between the two groups (* *p* < 0.05).

Salivary cytokine levels were compared between the two groups using the Mann–Whitney *U* test. The levels of IL-6, IL-8, and TNF-α on D1 were significantly higher in the Moderate to Severe group (*p* = 0.003, 0.001, and 0.007, respectively). Similarly, on D7, IL-6, IL-8, TNF-α, and VEGF levels were significantly elevated in the Moderate to Severe group (*p* = 0.001, 0.015, 0.045, and 0.001, respectively) (Table 2, Figure 2).

**Figure 3:**
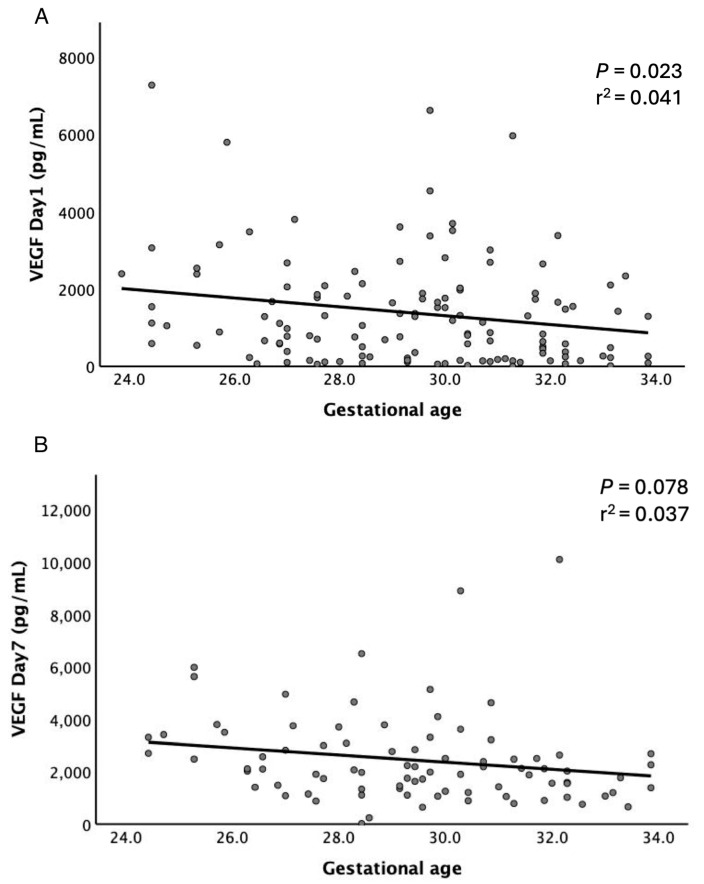
Correlations between GA and VEGF Levels. Distribution plots illustrate salivary VEGF levels on D1 (**A**) and D7 (**B**) among neonates with varying GAs. Solid lines depict the correlations, along with the corresponding *p* values and *r^2^* values.

As shown in Table 1, lower GA and birth body weight were significantly related to the severity of ROP. As noted in our previous study, salivary IL-6 and IL-8 were significantly negatively correlated with the premature neonates’ GA [22]. Thus, linear regression was employed to estimate the correlation between GA and VEGF levels. And VEGF levels exhibited a decreasing trend in relation to GA on both D1 and D7, with a particularly significant correlation on D1 (*p* = 0.023, *r^2^* = 0.041) (Figure 3). GA has a strong correlation with BW. Including both GA and BW in a logistic regression model may lead to an underestimation of the results due to multicollinearity. Therefore, additional analyses were performed using a multivariable logistic regression model, with adjustments made exclusively for neonatal GA. This analysis revealed that the levels of IL-6 and VEGF on D7 were significantly elevated in the Moderate to Severe ROP group compared to the None to Mild ROP group (*p* = 0.037 and 0.016, respectively) (Table 2).

## 4. Discussion

This study is the first to utilize salivary cytokines as biomarkers to predict the severity of ROP in premature infants. We found significantly higher levels of IL-6 and VEGF on D7 in the Moderate to Severe ROP group compared to the None to Mild ROP group. These findings suggest that the early postnatal measurement of specific cytokines in saliva, a non-invasive approach, could effectively predict the development of ROP.

ROP generally progresses through two distinct stages [4]. The first stage is characterized by the inhibition of normal retinal vascular development and maturation, while the second stage involves abnormal vascular proliferation. Recent studies have indicated that oxidative and nitrosative stress, along with inflammatory processes, play a significant role in the early phase of ROP [4]. Although the exact mechanisms by which these cytokines contribute to ROP development remain unclear, a potential hypothesis suggests that systemic inflammation may increase the vulnerability of the retina to hypoxic–ischemic injury or disrupt the balance between pro-angiogenic and anti-angiogenic factors, leading to abnormal blood vessel development [26]. Our study identified elevated levels of VEGF in early life as being strongly correlated with ROP severity.

VEGF is a key mediator of angiogenesis and is closely linked to the abnormal vascular proliferation observed in the second phase of ROP. Elevated VEGF levels have been detected in the vitreous fluid of patients with severe ROP [8,9], which is consistent with our findings. In our study, the VEGF levels in saliva on D7 were higher in infants in the Moderate to Severe ROP group. While vitreous fluid is a biofluid anatomically closer to the retina, its collection is more invasive than saliva. Notably, elevated VEGF levels have also been observed in cord blood samples from severe ROP cases, suggesting that VEGF levels may rise even before birth [27]. However, other studies have reported conflicting results. For example, some studies have reported decreased VEGF levels in the tear fluid of progressive ROP patients [18], which could be attributed to differences in birth weight and GA compared to our cohort. Furthermore, serum VEGF levels did not exhibit an increasing trend during the first week post-birth and showed no significant difference between ROP and non-ROP infants [28]. Some researchers have reported lower VEGF levels in the cord blood of ROP patients [29]. These studies included infants with higher birth weights and GAs than our cohort or did not differentiate by ROP severity. Genetic factors may also contribute to ROP development. Certain polymorphic alleles have been found to be significantly more common in ROP infants requiring treatment [28,29,30,31].

Studies suggest that VEGF, IL-6, IL-8, and TNF-α levels in cord blood may not reliably predict ROP [16], likely due to small sample sizes and unaccounted variables affecting cytokine production. While cord blood reflects perinatal stress, postnatal blood or other sample types provide insight into the infant’s condition after birth. Perinatal infections or inflammations, both known risk factors for ROP, can elevate inflammatory cytokine levels in cord blood [2]. In our study, we excluded cases of perinatal infection, such as maternal sepsis, chorioamnionitis, and neonatal sepsis, to minimize confounding factors that could influence cytokine levels.

Previous studies have found that plasma levels of IL-6, IL-8, and TNF-α on the first day after birth trended higher in the group requiring ROP treatment [32]. IL-6, a cytokine with both pro-inflammatory and anti-inflammatory roles, has been associated with intra-amniotic infection and neonatal sepsis [33,34]. Our earlier research demonstrated that IL-6 levels in saliva can accurately detect bacterial infections in premature infants [21]. Other pro-inflammatory cytokines, such as TNF-α and IL-1β, have been detected in the hypoxic neonatal retina [35]. Holm et al. observed that elevated plasma levels of IL-8 and TNF-α during the first three weeks of life were closely linked to an increased risk of pre-threshold ROP [12]. However, our study found that IL-8 and TNF-α levels in saliva were not associated with ROP severity after adjusting for GA. These discrepancies may be attributed to differences in the timing and types of sample collection. Saliva offers a more non-invasive and convenient method for assessing ROP severity.

The correlation between gestational age (GA) and VEGF levels on Day 1 was significantly stronger than on Day 7 (*p* = 0.023 vs. 0.078). This disparity may be attributed to invasive procedures frequently performed within the first seven days of life, including intubation, surfactant administration, catheter placement, and oxygen-supported ventilation, which can influence cytokine levels and diminish their association with GA by Day 7. VEGF levels on Day 1, however, are likely to provide a more accurate reflection of the neonates’ initial physiological condition, demonstrating a decreasing trend with increasing GA. Consistently, as reported in our previous study [22], other salivary cytokines such as IL-6, IL-8, and TNF-α also exhibit a negative correlation with GA. This observed trend likely represents the physiological cytokine expression in preterm infants, highlighting their potential as reliable biomarkers for various diseases.

Saliva offers notable advantages over serum, primarily due to its non-invasive collection method and ease of use. Similar to serum, saliva contains a wide array of biological components, including hormones, antibodies, growth factors, enzymes, and microbes, as well as their metabolic products. Many of these components in saliva are derived from the bloodstream through passive diffusion, active transport, or extracellular ultrafiltration, making it a valuable medium for assessing physiological functions or pathological changes [36]. Compared to umbilical cord blood and amniotic fluid, saliva provides a better means of tracking postnatal cytokine level changes. Although tear fluid shares a similar composition with other exocrine secretions, saliva collection is more convenient and non-invasive. Our study observed elevated levels of IL-6 and VEGF in saliva on D7 in infants with Moderate to Severe ROP, consistent with findings from other studies using other body fluids. Moreover, studies indicate that pro-inflammatory cytokines such as IL-6 and TNF-α increase in saliva in response to acute stressors, further supporting their utility as markers of systemic inflammation [37].

Given the limited blood volume in preterm infants and the potential adverse effects of stress, there is growing support for the use of less invasive sampling methods. Elevated levels of inflammatory mediators, such as IL-6 and IL-8, in amniotic fluid have been linked to ROP progression [15]. Cord blood samples with elevated levels of IL-6 and C5a have also been proposed as independent markers for predicting severe ROP. Additionally, the angiotensin-to-VEGF ratio in tears may serve as a predictor for ROP occurrence [18].

Our study does have several limitations. The volume and timing of feeding may affect the analysis of inflammatory markers in saliva. To minimize this impact, the preterm infants were in NPO status on the first day, and on the seventh day, saliva samples were consistently collected before feeding. The inconsistent time intervals between surfactant administration and saliva collection can vary among patients, making it challenging to assess the potential impact accurately. The number of severe ROP cases in our cohort is relatively small, despite the inclusion of 186 premature infants. Larger studies are necessary to confirm our findings. Additionally, we did not collect blood samples at the same time or track serial changes to compare blood and saliva cytokine levels over time. Such data would provide a more comprehensive view of cytokine level changes in preterm infants with ROP. Further large-scale studies are needed to clarify the correlation between the levels of IL-6, TNF-α, and VEGF in saliva, serum, or ocular fluids (such as tears, aqueous humor, or vitreous humor).

## 5. Conclusions

Our research indicates that IL-6 and VEGF could serve as important early indicators for predicting ROP. Larger studies are warranted to better understand the variations in salivary cytokines in ROP patients and to assess their practical utility.

## Figures and Tables

**Table 1:** Demographic and medical characteristics of premature infants with None to Mild and Moderate to Severe ROP.

Characteristics	None to Mild ROP (*n*= 107)	Moderate to Severe ROP (*n*= 18)	*p*
GA (weeks)	30.0 (28.3–31.7)	26.4 (24.4–27.8)	**<0.001**
BW (gm)	1280 (970–1600)	790 (703–1038)	**<0.001**
Sex (F/M)	48/59	10/8	0.451
Delivery mode (N/C)	40/67	9/9	0.434
A/S 1	6.0 (4.7–7.0)	5.0 (2.0–6.3)	0.054
A/S 5	8.0 (7.0–9.0)	7.0 (6.0–8.0)	0.065
G	2.0 (1.0–3.0)	1.5 (1.0–3.0)	0.572
*p*	1.0 (1.0–2.0)	1.0 (1.0–1.3)	0.166
SBP (mmHg)	51.0 (45.0–55.0)	50.5 (41.5–56.3)	0.888
DBP (mmHg)	27.0 (24.0–32.0)	29.0 (22.0–35.0)	0.547
RR (times/min)	47.0 (40.0–58.0)	42.0 (31.8–57.0)	0.195
HR (times/min)	149.0 (140.0–156.0)	151.5 (138.0–158.0)	0.877
BT (°C)	36.0 (35.2–36.5)	35.9 (35.1–36.1)	0.132
Days of invasive ventilation	3 (0–7.0)	54 (4.3–76.5)	**<0.001**
Surfactant administration	61 (57)	14 (77.8)	0.078
IVH	28 (26.2)	3 (16.7)	0.558
PVL	6 (5.6)	2 (11.1)	0.324
BPD	55 (51.4)	17 (94.4)	**<0.001**
**Maternal Condition**			
Preeclampsia	17 (15.8)	1 (5.5)	0.467
GDM	2 (1.9)	1 (5.5)	0.547
Antepartum fever	5 (4.72)	3 (18.8)	0.089
Hours of PROM	0 (0–72)	0 (0–3.8)	0.221
Antepartum antibiotic	96 (89.7)	18(100)	0.363
Antepartum steroid	76 (71.0)	16 (94.1)	0.151

The continues values are presented as median (25th–75th) calculated by Mann–Whitney *U* test. Categorical data are presented as numbers (percentage), calculated by chi-squared or Fisher’s exact test. A/S 1: Apgar score at one-minute test, A/S 5: Apgar score at five-minute test, BPD: bronchopulmonary dysplasia, BT: body temperature, BW: birth weight, DBP: diastolic blood pressure, F/M: female/male, G: gravida, GA: gestational age, GDM: gestational diabetes mellitus, HR: heart rate, IVH: intraventricular hemorrhage, N/C: normal spontaneous delivery/ Cesarean section, P: para, PROM: premature rupture of membranes, PVL: periventricular leukomalacia, ROP: retinopathy of prematurity, SBP: systolic blood pressure.

**Table 2:** The levels of salivary cytokines in neonates with different ROP severity in univariable and multivariable analysis.

Cytokines	ROP Severity	Univariable Median (IQR)	*p*	Multivariable OR (95% C.I.)	*p* *
IL-6 (Day 1)	None to Mild Moderate to Severe	1070 (351–3750) 6335 (1579–16,041)	**0.003**	1.000 (1.000–1.000)	0.430
IL-8 (Day 1)	None to Mild Moderate to Severe	17,640 (5888–28,356) 32,945 (22,144–52,679)	**0.001**	1.000 (1.000–1.000)	0.647
TNF-α (Day 1)	None to Mild Moderate to Severe	44.5 (14.6–113.8) 136.5 (44.2–454.8)	**0.007**	1.000 (0.999–1.001)	0.601
VEGF (Day 1)	None to Mild Moderate to Severe	845.0 (4.1–1814.4) 1638 (579–2667)	0.077	1.00 (1.000–1.001)	0.295
IL-6 (Day 7)	None to Mild Moderate to Severe	313 (115–1161) 2363 (977–7288)	**0.001**	1.000 (1.000–1.000)	**0.037**
IL-8 (Day 7)	None to Mild Moderate to Severe	19,625 (7788–39,148) 35,645 (21,506–67,234)	**0.015**	1.000 (1.000–1.000)	0.132
TNF-α (Day 7)	None to Mild Moderate to Severe	181.4 (82.8–496.1) 457.5 (135.4–2673.0)	**0.045**	1.000 (1.000–1.000)	0.255
VEGF (Day 7)	None to Mild Moderate to Severe	1902 (1207–2730) 3065 (2524–4641)	**0.001**	1.000 (1.000–1.001)	**0.016**

In the multivariable analysis, *p* * was calculated after adjusting gestational age. OR: odds ratio, CI: confidence interval.

## Data Availability

The data presented in this study are available on request from the corresponding author. The data are not publicly available due to ethical considerations.
